# Lithotomy in a Very Young Boy

**Published:** 1850-09

**Authors:** 


### SURGERY.

*Lithotomy in a very young Boy.* (Under the care of MR. AVERY.)—We have often expressed the opinion, that the cause of the frequent formation of calculus in the children of the laboring classes would be a very interesting subject of inquiry for the pathologist. No doubt locality, food, and diathesis have a great deal to do with this pathological fact, and it would appear that diathesis is sometimes so strongly marked, that a child may be born either with a strong propensity to renal concretion, or with a calculus in the bladder.

The little patient upon whom Mr. Avery lately operated, affords an excellent illustration of congenital stone in the bladder. The boy is now only two years and a few months old; he had calculous symptoms from birth, and when first seen by Mr. Avery suffered very much from them.

The child belongs to agriculturists, living in Wiltshire; the mother has had several other children, none of whom were affected with any congenital malformation or disease ; they are all tolerably strong and healthy, and the parents who enjoy the advantage of a country life, and labor in the open air, are of a good constitution, and exempt from any gouty or lithic diathesis. No particular circumstances accompanied the child's birth, but soon after it, and whilst he was being suckled, the mother's attention was drawn to the colour of his urine, which she noticed to be darker than she had known it to be with her other children. She was soon struck, likewise, with a peculiar way the child had of putting his hand on the hypogastrium, when only a few months old, as if anxious to relieve pain in that region. The urine, in the meantime, became darker, and soon assumed the appearance of blood, which circumstance naturally very much alarmed the mother.

When the child was about one year old, symptoms of a still more serious kind occurred; the mother stated that the abdomen became suddenly tympanitic, and the scrotum "as black," as she forcibly expressed it, "as your hat." No urine was expelled whilst these symptoms were present: but the child, to whose aid a medical man had been called, was relieved by emollient applications, &c. It may be surmised that the irritation caused by the presence of the calculus must have been great when these congestive phenomena appeared, and it is probable that the stone had accidentally been so placed as to obstruct for a time the flow of the urine.

The child was sounded at various times in the country, the surgeons in attendance were in doubt about the case, and the little patient was sent to Mr. Avery, in order that the question might be solved.

On admission, Mr. Avery found the child in tolerable good health, but its sufferings were very great whenever micturition took place, which function was performed very often. The child, as is generally the case in this affection, was ever pulling the prepuce, the mother stating that he was, while at home, in the constant habit of doing so, and that it grieved her to see him suffer so much every time he evacuated the bladder. Mr. Avery having passed a small sound, ascertained the presence of the calculus; the striking of the instrument against the stone gave the impression that the latter was very small; and Mr. Avery bearing in mind the advantages of lithotomy over lithotrity in the case of children, resolved to perform the first of these operations.

On the 17th of June, the child was brought into the theatre, and having been rendered insensible by a piece of lint, sprinkled with chloroform, being lightly held over the face, Mr. Avery made the usual incisions, and cautiously extracted from the bladder a round stone about the size of a horse-bean. On examining it at first, it appeared strange that the symptoms should have been so distressing at the very onset of the affection, when the stone, as it fairly may be supposed, was considerably smaller. Still foreign bodies, however small, will cause disturbance according to the susceptibility of the organ where they happen to be placed, and the age of the patient.

The child lost very little blood, and was removed, while still under the influence of chloroform. The progress of the little patient was extremely favorable; three days after the operation, the urine passed almost entirely per urethram; but in a few days it again flowed almost wholly through the wound, with considerable pain and irritation about the part. This change was, however, of short duration; and six days after the extraction of the stone, the urine again passed through the urethra, and gradually was entirely evacuated by the natural passage, the wound being quite cicatrised. On the nineteenth day the part was perfectly closed, and the boy left the hospital quite restored, about five weeks after admission. The stone was examined by Mr. John Quekett, and found composed of a nucleus of oxalate lime, invested by a layer of phosphates.

This case affords some confirmation to the opinion that children may be visited by any disease in utero, as it would appear that in this instance the patient was affected with stone, either in the kidney or bladder, before he saw the light. Such cases likewise show the necessity of giving proper attention to apparently trifling symptoms in children: frequency of micturition, occasional pain in evacuating the bladder, the habitual pressure of the hand on the hypogastrium, should not be overlooked, especially in boys, and suspicions be excited, that these symptoms *may* be owing to a congenitally-formed calculus. It may likewise be observed that if the early symptoms had suggested the employment of an instrument, and a slight dilatation of the urethra at an early period, it is more than probable that the calculus, in its then small state, would have been evacuated.—*London Lancet.*

